# Selective angiography through radiofrequency catheter during ablation of premature ventricular contractions originating from aortic cusp: A single-centre experience

**DOI:** 10.1016/j.ipej.2022.03.005

**Published:** 2022-04-04

**Authors:** Umut Celikyurt, Burak Acar, Irem Karauzum, Kaan Hanci, Ahmet Vural, Aysen Agacdiken

**Affiliations:** Kocaeli University Medical Faculty, Arrhythmia, Electrophysiology, Pacemaker Research and Management Center, Kocaeli, Turkey

**Keywords:** Premature ventricular contraction, Aortic cusp, Coronary angiography

## Abstract

**Introduction:**

Intraprocedural coronary angiography is recommeded in patients undergoing ablation in aortic cusps to assess the relation of catheter tip and coronary ostia. In this report, we present our experience in selective coronary angiography through the radiofrequency catheter during premature ventricular contraction (PVC) ablation.

**Methods and results:**

We prospectively recruited 43 consecutive patients who underwent PVC ablation in the aortic cusps between March 2018 and April 2021. We performed coronary angiography through the contact force (CF)-sensing ablation catheter at the ablation site. Successful ablation was achieved in 38 (88%) of patients. No technical problems occurred after the contrast injection and ablation parameters were within the normal values, without any change of impedance and CF-sensing values and neither electrogram signal quality after contrast injection. No complications occurred during the procedure, hospital stay, and during one-year follow-up (15.3 ± 3.1 months).

**Conclusion:**

Selective coronary angiography through the CF-sensing ablation catheter to assess the relation between the ablation site and the coronary ostia is feasible and no minor or major complications occurred in our experience.

## 1 Introduction

Radiofrequency catheter ablation (RFCA) is an effective and feasible technique for treating patients with ventricular arrhythmia (VA) originating from the outflow tract. Approximately 15% of idiopathic VAs originates from the left ventricular outflow tract (LVOT) [1]. RFCA can be safely performed in the VAs originating from OTs [2].

Coronary angiography is recommended during ablation within the coronary cusps to estimate the distance from catheter tip to coronary arteries to avoid the potential damage to coronary arteries [3]. Ablation is considered safe when the distance between ablation site and coronary ostium is over 5 mm [4]. However, coronary angiography is an invasive procedure, requires an additional vascular access and also increases the risk of complications.

Recently**,** Roca-Luque et al. was the first who reported the feasibility of selective angiography through a cooled-tip radiofrequency ablation catheter as an alternative technique to assess relation of coronary ostia and aortic cusp ablation site [5]. In this report, we aimed to present our experience, in terms of effectiveness and safety of selective coronary angiography through the radiofrequency catheter during premature ventricular contraction (PVC) ablation.

## 2 Methods

### 2.1 Patients and study protocol

The study cohort comprised 43 consecutive patients who underwent ablation in the aortic cusps in our department between March 2018 and April 2021. Data were consecutively collected case-by-case and entered into a computerized database.

All patients signed and informed consent with specific details about this technique. The study was approved by the institutional ethics review board.

### 2.2 Electrophysiological study

Antiarrhythmic drugs were discontinued for a period of at least five half-lives before electrophysiological study. Electroanatomic mapping (CARTO III, Biosense Webster, Diamond Bar, CA, USA) was used to guide aortic cusp ablation in all cases. Right side femoral vein and/or artery were gained for mapping and ablation. Twelve-lead surface electrocardiograms (ECGs) and intracardiac electrograms were recorded simultaneously on an electrophysiological recording system (Prucka CardioLab, GE Healthcare, Waukesha, WI). In cases with infrequent PVC, isoproterenol was injected (1–20 μg/min) to promote triggered activity and manifest PVCs. A retrograde aortic approach to map and ablate in the aortic cusps was used. A 3.5-mm-tip irrigated contact force (CF)-sensing catheter (SmartTouch™, Biosense Webster, Inc, Diamond Bar, CA) was used in all cases for mapping and ablation. After gaining artery access, intravenous unfractionated heparin was given to all patients and titrated to maintain an activated clotting time of >300 s. Site of origin was determined as that with the earliest activation and/or best pace mapping (PASO score ≥95%) as demonstrated by automatic pace-mapping algorithm (PASO, CARTO III, Biosense Webster, USA). Low-osmolar contrast media (iopamidol, Iopamiro™ 300 mg iodine/mL) was flushed manually (less than 4 cc were needed in every injection) through the ablation catheter immediately before the delivery of radiofrequency ablation aiming to rule out the presence of coronary arteries or to assess the proximity of the catheter tip to the coronary ostium in case of left or right coronary cusp (LCC, RCC) in all cases. Detailed visual examination during manual injection of contrast media and automatic saline irrigation pump during all the procedure were used in order to avoid air embolism. The heparin saline flush post contrast use was done to avoid any clots. Normal flow (4 cc/min irrigation) heparinized saline was resumed after injection. Radiofrequency with energy titration (20–30 W, 43 °C, 17 cc/min irrigation) was delivered depending on the distance from the coronary ostium, impedance drop, tip temperature, and response to ablation [6]. Radiofrequency application will be terminated if PVCs did not disappear within 5–10 s or the catheter was dislodged. When elimination of PVCs was seen within 5s of onset of ablation, power delivery was stopped after 30s. Ablation was not attempted at sites with a distance <5 mm. Continuous impedance, ECG monitoring during all procedures was performed.

Acute success and acute and 30-day complications and one-year follow-up data were recorded in a prospective database. Successful ablation was defined as the complete elimination of the PVC focus at the end of a 30-min waiting period. Long-term ablation success was defined as a decrease >95% in PVC burden at 1-year Holter monitoring. All patients were underwent a transthoracic echocardiogram at the end of the procedure to rule out pericardial effusion.

## 3 Results

A total of 43 consecutive patients were included in case series. All of the patients presented with PVC, and 4 patients (9%) had previous failed ablation. Patients had a high PVC burden (17.9 ± 5.4%), five patients had dilated cardiomyopathy and four patients had a history of coronary heart disease. The mean age was 52 ± 18 years, with the majority being men (26 of 43 [61%]).

**Fig. 1:**
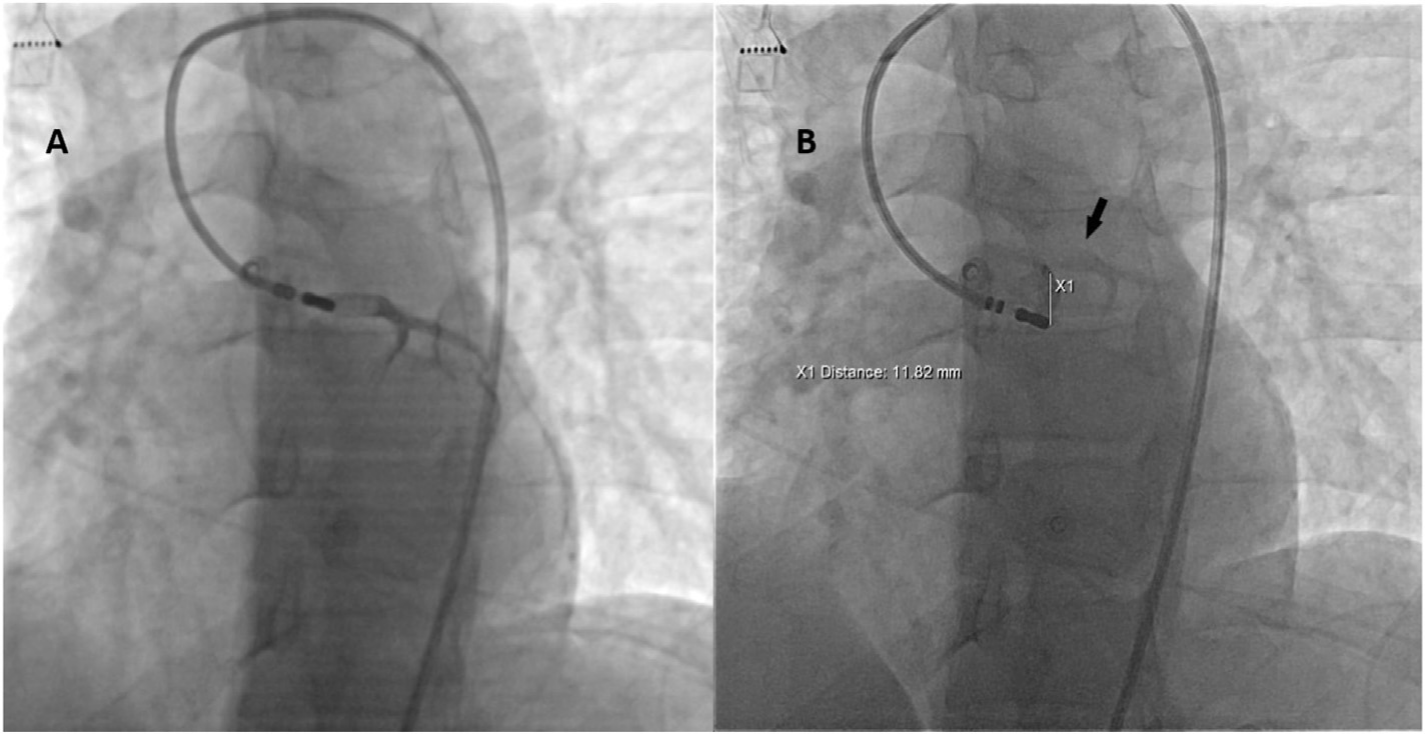
**A** LAO fluoroscopy projection. Selective angiography in LCC showing left main coronary artery, **B** Selective angiography of the same patient showing enough distance from catheter tip to left main coronary artery (black arrow) to deliver RF energy in the earliest activation site.

**Fig. 2:**
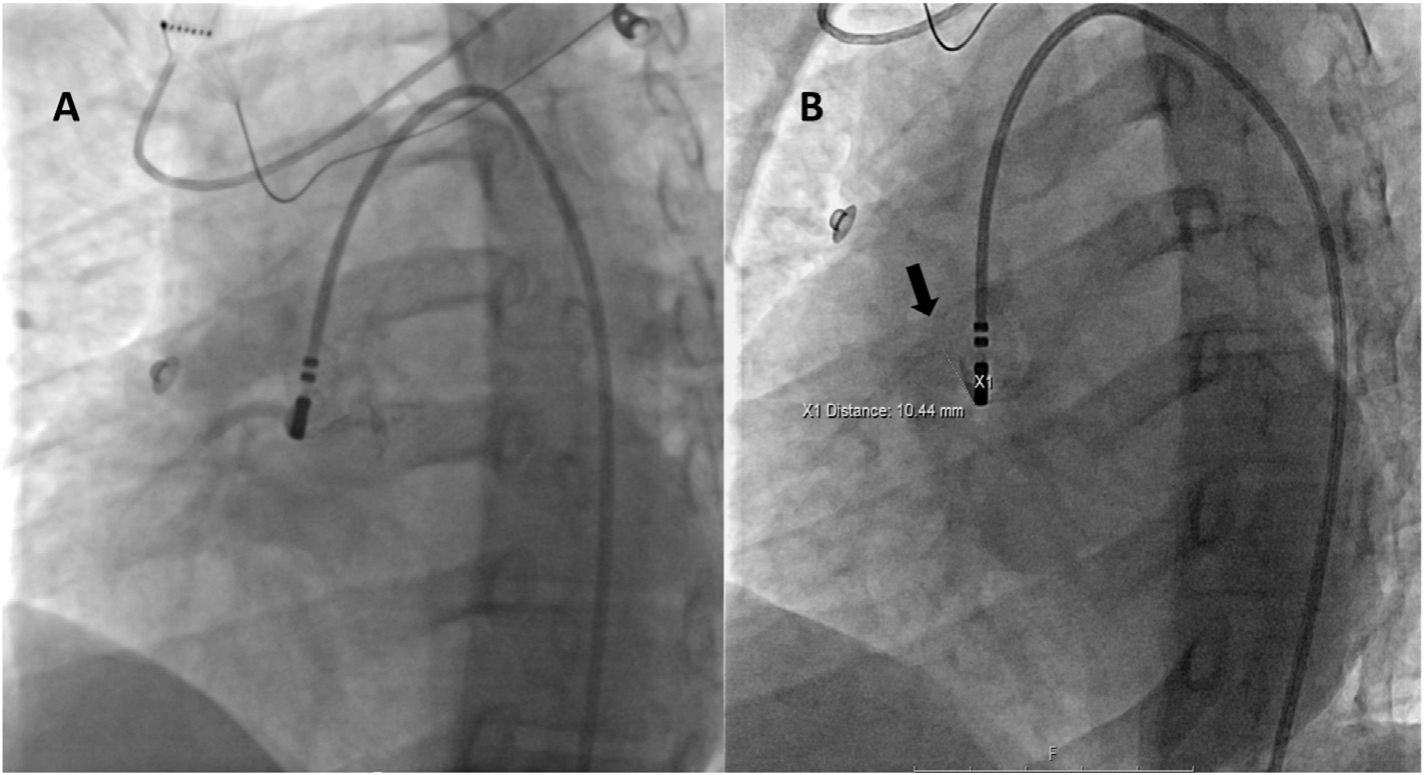
**A** LAO fluoroscopy projection. Selective angiography in RCC showing right coronary artery, **B** Selective angiography of the same patient showing enough distance from catheter tip to right coronary artery ostium (black arrow) to deliver RF energy in the earliest activation site.

The local electrogram onset was 23.7 ± 8.1 ms before QRS onset. Successful ablation was achieved in 38 (88%) of patients. The left cusp was the site of PVC origin in the majority of patients (33 of 43 [77%], followed by the right cusp (7 of 43, [16%]), left-right cusp junction (3 of 43, [7%]). In all cases, selective coronary angiography documented a distance of >10 mm from the coronary ostia and ablation was performed (Fig. 1, Fig. 2).

**Fig. 3:**
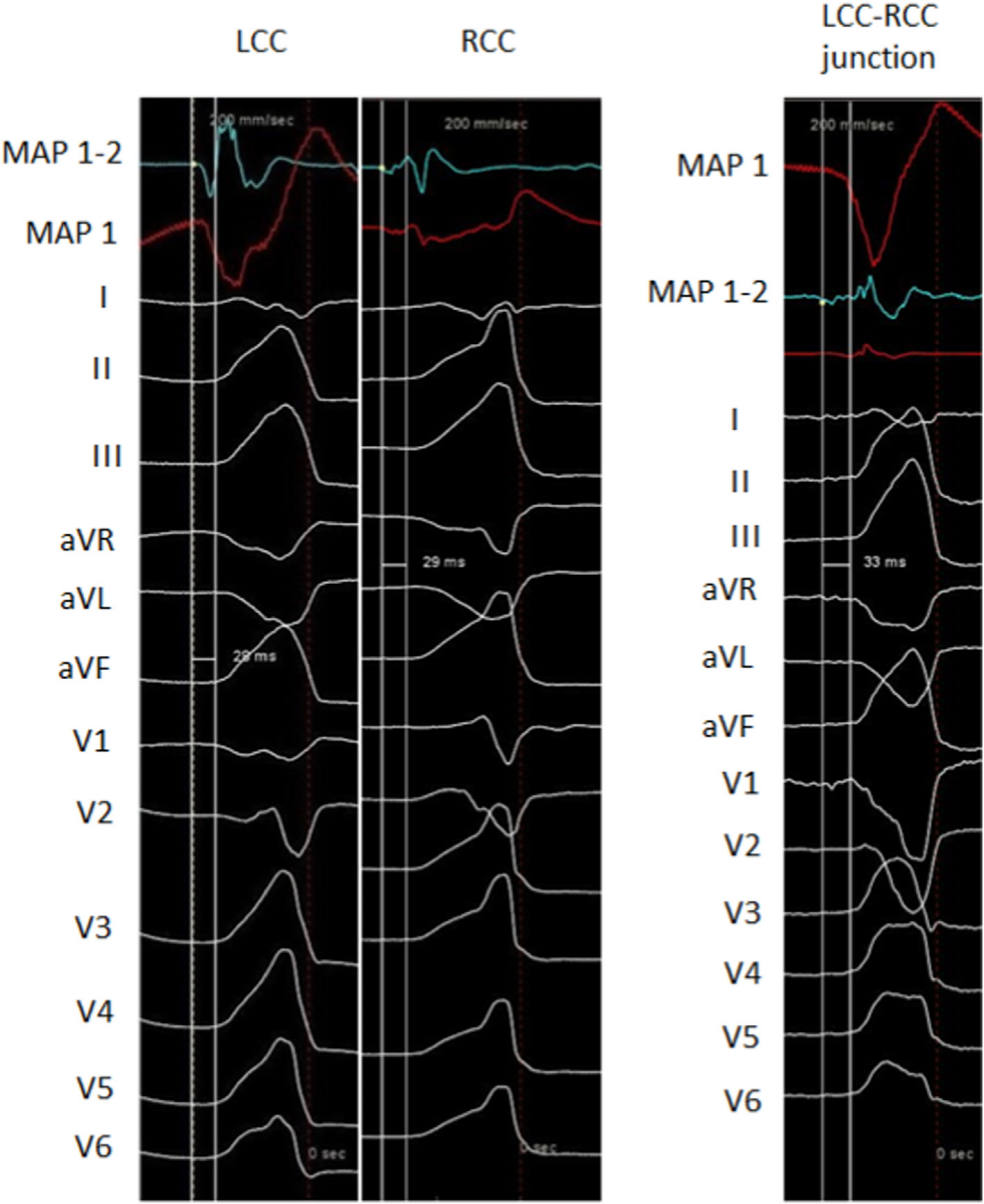
12-lead electrocardiogram of PVCs originating from LCC, RCC and LCC-RCC junction, along with earliest bipolar and unipolar activation time at successful ablation site.

In 35% of patients right ventricular outflow tract (RVOT) was mapped first and in 21% of patients both RVOT and LVOT ablated. The mean CF at the site of successful ablation was 11 ± 2 g. Fig. 3 shows earliest bipolar and unipolar activation time at successful ablation site of PVCs from LCC, RCC and LCC-RCC junction. The mean number of RFA deliveries and total RFA duration were 12.7 ± 9.3 and 76.1 ± 43.0 s, respectively. The mean total procedural time was 96 ± 21 min.

**Fig. 4:**
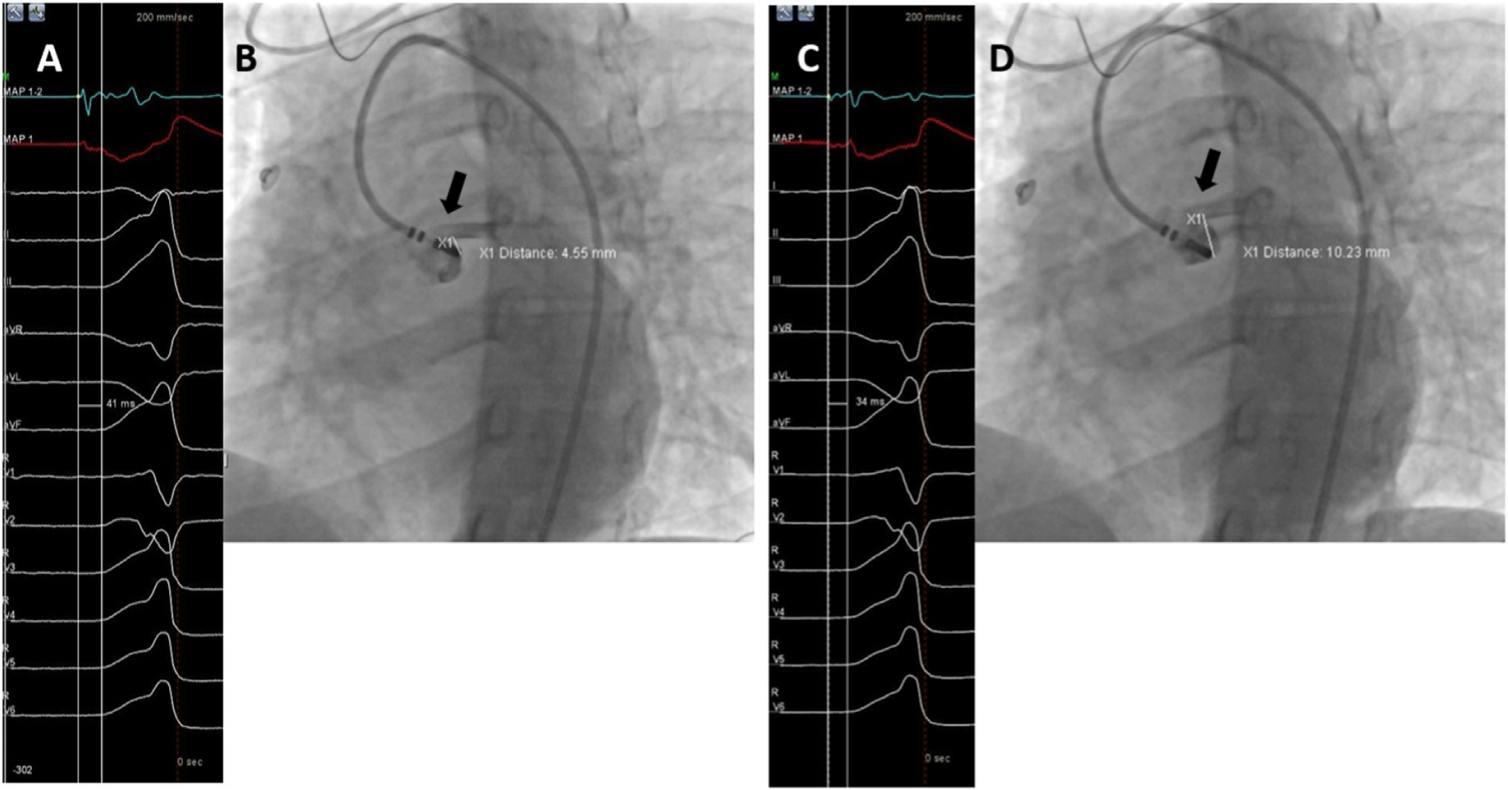
**A** 12-lead electrocardiogram of PVC at earliest site of ventricular activation (preceding onset of the QRS wave by 41 ms on surface electrocardiogram), **B** selective angiography showing not enough distance (<5 mm) from catheter tip to left main coronary artery (black arrow) to deliver RF energy in the earliest activation site. The catheter was repositioned to the closest safe site maintaining promising activation time. **C** 12-lead electrocardiogram of PVC at promising site of ventricular activation (preceding onset of the QRS wave by 34 ms on surface electrocardiogram), **D** repeated selective angiography showing enough distance (>5 mm) from catheter tip to left main coronary artery (black arrow), and RFA was delivered.

In 36 patients (84%) coronary artery visualization was again required after catheter repositioning due to failed ablation attempts or too short distance between the coronary ostia and the earliest site of ventricular activation (Fig. 4). No technical problems occurred after the contrast injection and ablation parameters were within the normal values, without any change of impedance and CF-sensing values and neither electrogram signal quality after contrast injection. No complications occurred during the procedure, hospital stay, and during follow-up (15.3 ± 3.1 months).

At 1-year follow-up 38 of patients (88%) showed a decrease >95% in PVC burden at Holter monitoring.

## 4 Discussion

In this single-center case series, we have shown that selective coronary angiography through the radiofrequency catheter during PVC ablation is the feasible and effective. Roca-Luque et al. first described the feasibility of selective coronary angiography through a cooled-tip radiofrequency ablation catheter in 12 consecutive patients [5]. Half of their patient population underwent ablation in right and left coronary cups. Our experience, on a larger sample, confirms the feasibility and safety of this technique without any major or minor adverse events during one-year follow-up period.

Catheter ablation in the aortic cusps can be challenging owing to the complex anatomic relationships of the aortic valve, coronary arteries, and veins. Coronary angiography during ablation is used to estimate distance from catheter tip to coronary arteries to avoid potential damage to coronary arteries. However, coronary angiography presents risks, including air embolism and coronary dissection [7,8]. In addition, a second arterial puncture is necessary to evaluate the ablation catheter tip with relation to the coronary ostia in a single image in case of PVCs originating from aortic cusps, which might increase the frequency of complications at the access site. Selective coronary angiography through ablation catheter allows real-time mapping and ablation in aortic cusps and avoids need for an additional arterial access.

Also, with selective coronary angiography repeated injections can be performed at the ablation site when repositioning of the catheter is needed. One of the advantages of doing selective coronary angiography through the ablation catheter is that the ostium has been tagged and the distance from the new point to the ostium was visible and real-time relation of the catheter tip and coronary ostium is always assessed. In our case series, coronary artery visualization was again required in 84% of patients after catheter repositioning due to failed ablation attempts or too short distance between the coronary ostia and the earliest site of ventricular activation. This high percentage of repeated coronary angiography may be related to easily performed coronary angiography through the ablation catheter during the ablation procedure. Although the ostium was tagged on 3D mapping, we did coronary angiography through ablation catheter at the new ablation site to confirm the distance of the ostium from the ablation site. In fact, post ablation coronary angiography can be done as a routine to document for any damage to the vessel even when the ablation was done away.

Hoffmayer et al. investigated intracardiac echocardiography (ICE) guidance for the aortic root VAs ablation without aortography or coronary angiography [9]. They showed that ICE could be used to assess real-time distance between catheter tip and coronary ostium. In their study, coronary angiography was required in 10% of the patients to confirm catheter tip distance to the coronary artery, and repeated coronary angiography is necessary to assess real-time relation of catheter tip and the coronary ostium after failed ablation. Recently, Asmar et al. confirmed the safety of mapping in the sinus of valsalva region under ICE guidance without coronary angiography [7]. However, any additional invasive technique increases the risk of procedure-related complications. Also, ICE is an expensive modality and could not be routinely used in all patients, especially in low income countries.

Radiofrequency catheter ablation is a safe and effective treatment for patients with symptomatic PVC originating from the outflow tract, but also carries risks because of proximity to the coronary ostium. Jagadheesan et al. investigated the outcomes with low power ablation in left coronary cusp, and their results suggest that higher powers may not be necessary for aortic cusp ablations, and ablation with low power up to 30 W is effective in achieving acute and chronic success while avoiding complications [6]. In our case series, we also used radiofrequency with energy titration up to 30 W to reduce the risk of coronary artery injury.

In 21% cases where RVOT was also ablated, radiofrequency ablation failed to suppress PVCs despite the local ventricular activation preceding the QRS onset of the PVCs. Outflow tract anatomy and electrophysiological properties of the surrounding myocardium may explain the reason for failed ablation. Specialized myocardial fibers can contribute to preferential conduction from the aortic sinus cusps to the RVOT [10,11]. Therefore, we mapped aortic sinus cusps after failed RVOT ablation instead of increasing the power.

In addition, Roca-Luque et al. suggested that bigger pore size may effect the quality of the angiography and reported that better images were obtained with 7F Therapy™ Cool Flex™ ablation catheter [5]. In our experience, 8F SmartTouch™ catheter was used, which enables us to obtain good image quality, probably because of one French bigger size. Therefore, in patients undergoing PVC ablation, 8F catheters could be more feasible, if selective coronary angiography through the ablation catheter is planned.

Contrast injection through the ablation catheter has not been widely studied, and the safety of this method is not well known. Roca-Luque et al. reported a 12 patient series using contrast injection through the ablation catheter without any technical difficulties in any patient [5]. Although contrast injection through the ablation catheter is not described in the technical specifications of radiofrequency ablation catheters, in our case series catheter performance, impedance and CF-sensing values and electrogram signal quality were not changed after contrast injection. In addition, the heparin saline flush post contrast use should be done to avoid any clots. Also, we finished all the procedures with one ablation catheter.

## 5 Conclusions

Selective coronary angiography through the CF-ablation catheter to assess the relation between the ablation site and the coronary ostia is feasible and no minor or major complications occurred in our experience. 8F catheters could be more appropriate if intraprocedural selective coronary angiography is planned. Continued investigation is required to evaluate success and complication rates.

## Limitations

This is a single-center study and only one catheter type was used in the study. Our results could not be generalized to all CF-catheters. In addition, selective coronary angiography through the ablation catheter does not allow high definition angiography. However, the aim of selective coronary angiography is not to visualize the coronary arteries, but to assess the distance between the catheter tip and coronary arteries to avoid the potential damage to the coronary arteries. Selective coronary angiography revealed acceptable image quality to assure the safety distance.

## Funding

None.

## Author contributions

UC, AA, and BA designed and prepared the manuscript. UC, AA, and AV performed the procedures. BA and KH collected the data. UC and IK reviewed the manuscript draft.

## Declaration of competing interest

None declared.
